# Impact of HLA Mismatching on Early Subclinical Inflammation in Low-Immunological-Risk Kidney Transplant Recipients

**DOI:** 10.3390/jcm10091934

**Published:** 2021-04-29

**Authors:** Domingo Hernández, Teresa Vázquez, Juana Alonso-Titos, Myriam León, Abelardo Caballero, María Angeles Cobo, Eugenia Sola, Verónica López, Pedro Ruiz-Esteban, Josep María Cruzado, Joana Sellarés, Francesc Moreso, Anna Manonelles, Alberto Torio, Mercedes Cabello, Juan Delgado-Burgos, Cristina Casas, Elena Gutiérrez, Cristina Jironda, Julia Kanter, Daniel Serón, Armando Torres

**Affiliations:** 1 Nephrology Department, Hospital Regional Universitario de Málaga, University of Málaga, Instituto de Investigación Biomédica de Málaga (IBIMA), REDinREN (RD16/0009/0006), E-29010 Málaga, Spain; Teresavs89@hotmail.com (T.V.); juana12041988@hotmail.com (J.A.-T.); sola.moyano.eugenia@gmail.com (E.S.); verolopezjim@yahoo.es (V.L.); pedro_ruiz_esteban@hotmail.com (P.R.-E.); mcabello82@hotmail.com (M.C.); juandelgadob@gmail.com (J.D.-B.); cristinacasasgonzalez@gmail.com (C.C.); egutierrezvilchez@gmail.com (E.G.); cristinajironda@gmail.com (C.J.); 2 Pathology Department, Hospital Regional Universitario de Malaga, Instituto de Investigación Biomédica de Málaga (IBIMA), REDinREN (RD16/0009/0006), E-29010 Málaga, Spain; mlfradejas@gmail.com; 3 Immunology Department, Hospital Regional Universitario de Málaga, University of Málaga, Instituto de Investigación Biomédica de Málaga (IBIMA), REDinREN (RD16/0009/0006), E-29010 Málaga, Spain; abelardo.caballero.g@gmail.com (A.C.); alberto.torio@gmail.com (A.T.); 4 Nephrology Department, Instituto de Tecnologías Biomédicas-Universidad La Laguna, Hospital Universitario de Canarias, REDinREN (RD16/0009/0031), E-38320 Tenerife, Spain; mcobcas@gmail.com (M.A.C.); atorresram@gmail.com (A.T.); 5 Nephrology Department, Hospital Universitari de Bellvitge, University of Barcelona, IDIBELL, REDinREN (RD16/0009/0003), E-08907 Barcelona, Spain; jmcruzado@bellvitgehospital.cat (J.M.C.); amanonelles@bellvitgehospital.cat (A.M.); 6 Nephrology Department, Hospital Universitari Valld’Hebron, Universitat Autonoma, Barcelona, REDinREN (RD16/0009/0030), E-08035 Barcelona, Spain; jsellares@vhebron.net (J.S.); fjmoreso@vhebron.net (F.M.); dseron@vhebron.net (D.S.); 7 Nephrology Department, Hospital Universitario Dr. Peset, E-46017 Valencia, Spain; julikanter@gmail.com

**Keywords:** kidney transplantation, HLA compatibility, subclinical inflammation, Banff criteria, low-immunological risk

## Abstract

The impact of human leukocyte antigen (HLA)-mismatching on the early appearance of subclinical inflammation (SCI) in low-immunological-risk kidney transplant (KT) recipients is undetermined. We aimed to assess whether HLA-mismatching (A-B-C-DR-DQ) is a risk factor for early SCI. As part of a clinical trial (Clinicaltrials.gov, number NCT02284464), a total of 105 low-immunological-risk KT patients underwent a protocol biopsy on the third month post-KT. As a result, 54 presented SCI, showing a greater number of total HLA-mismatches (*p* = 0.008) and worse allograft function compared with the no inflammation group (48.5 ± 13.6 vs. 60 ± 23.4 mL/min; *p* = 0.003). Multiple logistic regression showed that the only risk factor associated with SCI was the total HLA-mismatch score (OR 1.32, 95%CI 1.06–1.64, *p* = 0.013) or class II HLA mismatching (OR 1.51; 95%CI 1.04–2.19, *p* = 0.032) after adjusting for confounder variables (recipient age, delayed graft function, transfusion prior KT, and tacrolimus levels). The ROC curve illustrated that the HLA mismatching of six antigens was the optimal value in terms of sensitivity and specificity for predicting the SCI. Finally, a significantly higher proportion of SCI was seen in patients with >6 vs. ≤6 HLA-mismatches (62.3 vs. 37.7%; *p* = 0.008). HLA compatibility is an independent risk factor associated with early SCI. Thus, transplant physicians should perhaps be more aware of HLA mismatching to reduce these early harmful lesions.

## 1. Introduction

Although new immunosuppressant drugs have led to a reduction in acute rejection rates after kidney transplantation (KT) over recent years, this improvement has not translated into longer allograft survivals, suggesting that the alloimmune response phenomenon could be a crucial risk factor implicated in long-term graft loss [1,2,3].

Low-grade graft inflammation not qualifying as rejection according to revised Banff criteria [4], i.e., subclinical inflammation (SCI) or borderline lesions (BL), is very common post-transplantation (≈50%), even during the first months post-transplantation. This suggests that inflammatory phenomena could initiate soon after KT and, thereafter, perpetuate in shaping chronic changes in the kidney allograft histology, leading to long-term graft loss [5,6,7,8,9]. However, evidence is lacking on clinical risk factors associated with early SCI after KT in the modern transplant era [10,11,12]. 

Understanding risk factors for graft loss and advances in immunosuppression have increased the debate over the years about the true role of human leukocyte antigen (HLA) compatibility in kidney graft survival. Indeed, while some have shown that both the number of HLA-mismatches and the particular molecular HLA-mismatches involved adversely influence graft survival [12,13,14,15,16,17,18,19,20,21], others have not [22,23,24,25], arguing for a diminishing significance of HLA over the more recent transplant era. Nevertheless, the effect of HLA compatibility on the appearance of SCI in KT recipients during the first three months post-KT, where other immune response-related factors concur, is still uncertain. 

The purpose of this study, therefore, was to assess the impact of HLA mismatching on early (3rd month) SCI in low-immunological-risk KT recipients in daily clinical practice, a setting where a proper HLA match prior to KT is often overlooked and where other factors involved in modulating the immune response, such as induction therapy, delayed graft function, tacrolimus levels, or pretransplant transfusions, could be present.

## 2. Material and Methods

This study is part of an investigator-driven, parallel-group, open-label-multicenter, randomized clinical trial undertaken in low-immunological-risk KT recipients comparing corticosteroid withdrawal versus standard immunosuppression (number NCT02284464, https://clinicaltrials.gov/, accessed on 27 April 2021). We studied a total of 105 Caucasian KT patients from 5 Spanish transplant centers located in Malaga, Tenerife, and Barcelona. The patients underwent a protocol biopsy 3 months post-KT immediately prior to randomization according to the previously described study design. No patient had biopsy-proven rejection during the first three months post-KT, and all had stable graft function, as well as the absence of de novo donor-specific antibodies (dnDSA) at the time of the protocol biopsy, using an MFI cut-off level of 500 U (One Lambda LABScreen-single antigen bead assay). All the patients gave informed consent, and the study was approved by the ethics and clinical research committee of each participating center, as well as by the Spanish Drug Agency (EudraCT 2012-003298-24). The study followed the principles of the Declarations of Helsinki and Istanbul.

HLA typing was performed in donor and recipients by the polymerase chain reaction sequence-specific oligonucleotide method (PCR-SSO) combined with Luminex technology, carried out using a LABType RSSOH2B1 (HLA-HD) commercial kit (One Lambda, Inc., West Hills, CA, USA). The protocol comprised the DNA amplification process using the PCR-SSO method, reading on a special device (LABScanTM3D) and software interpretation (HLA Fusion^TM^) following the manufacturer’s instructions.

Immunosuppression consisted of induction therapy (basiliximab or thymoglobulin), according to the protocol of each participating center, plus 0.5 g of methylprednisolone intravenously intraoperatively and 125 mg on day 1; 30 mg/day of prednisone for the first four days with gradual dose reduction until reaching 5 mg/day in the second month post-transplantation. In addition, 0.15 mg/kg p.o. per day of tacrolimus (TAC) (Prograf^®^ (Astellas Pharma Tech Co., Toyama, Japan) or Advagraf^®^ (Astellas Ireland Co., Killorglin, Ireland)) was administered to maintain trough levels of 8–12 ng/mL on the first month and, later, 0.1 mg/kg/day of TAC (trough levels of 5–8 ng/mL). Furthermore, 2 g/day of MMF during the first 15 days post-transplantation and, later, 1 g/day of MMF plus 5 mg/day p.o. of prednisone were administered during the rest of the study up to randomization.

All protocol biopsies were performed at 3 months post-transplant as an outpatient procedure, and they were performed under ultrasound guidance using an 18 G spring-loaded biopsy needle. At least one core of tissue with a minimum of 7 glomeruli and one artery were required for proper interpretation. SCI, including BL and isolated mild inflammation without tubulitis (IIF) (i1, t0), was defined as an interstitial inflammation score (i) and/or tubulitis score (t) of at least 1, but below the threshold for Banff 1 A rejection (Banff i2, t2) [4]. Additionally, the chronic allograft histology score was obtained using a composite of chronic interstitial (ci), chronic tubular (ct), and chronic glomerular (cg) plus chronic vascular (cv) (ci + ct + cg + cv), as well as using a composite of interstitial fibrosis and tubular atrophy (IFTA) score (ct + ci). IFTA was defined as the sum of ci + ct ≥ 2 [7,26]. Experienced transplant pathologists interpreted all biopsies, and inflammatory and chronicity scores were all validated by a single pathologist (ML).

Patients were monitored clinically weekly during the first month and on the second and third month post-KT. The allograft function was estimated by MDRD-4 (GFR), and proteinuria was determined either in 24 h urine or as the protein: creatinine ratio in a first-morning voiding sample.

### Statistical Analysis

Quantitative variables are expressed as the mean ± standard deviation or as median and interquartile range (IQR), and qualitative variables as percentages. Statistical analysis was started by comparing the two study groups, defined by the presence or absence of SCI at the 3-month protocol biopsy. Inter-group comparisons of quantitative variables were made by the Student’s t-test or the Mann–Whitney U-test as appropriate. Categorical variables were compared using the chi-square test or Fisher’s exact test. Stepwise multiple logistic regression analysis was used to assess the relationship between SCI and HLA mismatches after adjusting for other confounding clinical variables. These variables included recipient age, donor age, mean TAC levels (considering five TAC levels before the protocol biopsy), induction therapy (basiliximab vs. thymoglobulin), expanded criteria donor, delayed graft function, defined as the need for dialysis in the first week post-transplantation, cold ischemia time, and transfusions prior to KT. These clinical variables have been related to acute graft inflammation [27,28,29,30,31]. The Hosmer–Lemeshow goodness of fit was the principal criterion for selection of the final models. Receiver operating characteristic (ROC) curves were plotted and the area under the curve (AUC) was estimated to assess the predictive performance of total HLA mismatches and class II HLA mismatches in discriminating SCI. In addition, the best discrimination limit for HLA-mismatch levels was explored by the maximum of the Youden’s index (sensitivity + specificity-1). This corresponds to the value where sensitivity plus specificity is maximized. Finally, patients were clustered into two groups according to the Youden’s index in order to assess the clinical and histological characteristics in both groups. Analyses were done with SPSS 20.0 (IBM SPSS statistic). A *p* < 0.05 was considered significant. 

## 3. Results

Table 1 displays the baseline clinical data of the patients at three months post-transplantation. No patient had panel reactive antibodies prior to KT. Half the patients had ≥6 HLA mismatches, and the median number of total HLA mismatches was 6 (IQR 5–8). 

The histological study showed that 51 patients had no inflammation (NI) and 54 had SCI (including IIF, *n* = 22). As expected, patients with SCI had significantly higher acute inflammation and chronicity scores despite comparable TAC levels (Table S1). A significantly greater proportion of patients with SCI had IFTA ≥ 2 compared to those with NI (45.8 vs. 25%; *p* = 0.037). Importantly, patients with SCI had a significantly higher median number of class II HLA mismatches (DR-DQ) and total HLA mismatches (A-B-C-DR-DQ) compared with the NI group. These differences were also seen when patients with IIF were excluded (median of total HLA-mismatches: 5.5, IQR 4–7 vs. 7, IQR 6–9; *p* = 0.002). Finally, a worse allograft function was observed in patients with SCI (Table 1).

Table 2 shows the relationship between total HLA mismatches and SCI after adjustment for confounder variables such as recipient and donor age, delayed graft function, induction therapy, expanded criteria donor, transfusion prior to KT, and mean TAC levels. Of note, an increase in only one HLA-antigen mismatch was associated with a 32% increase in SCI risk. Similarly, class II HLA mismatching was independently associated with SCI (Table 2). When patients with IIF were excluded from the analysis, a similar relationship between HLA mismatches and SCI was also seen after adjusting for the same confounder variables (OR 1.51, 95%CI 1.11–2.04; *p* = 0.008). By contrast, class I HLA mismatches was not associated with SCI in the multivariate regression model. Finally, when individual class II HLA-loci were analyzed, HLA-DR mismatches and HLA-DQ mismatches were not associated with SCI.

**Figure 1:**
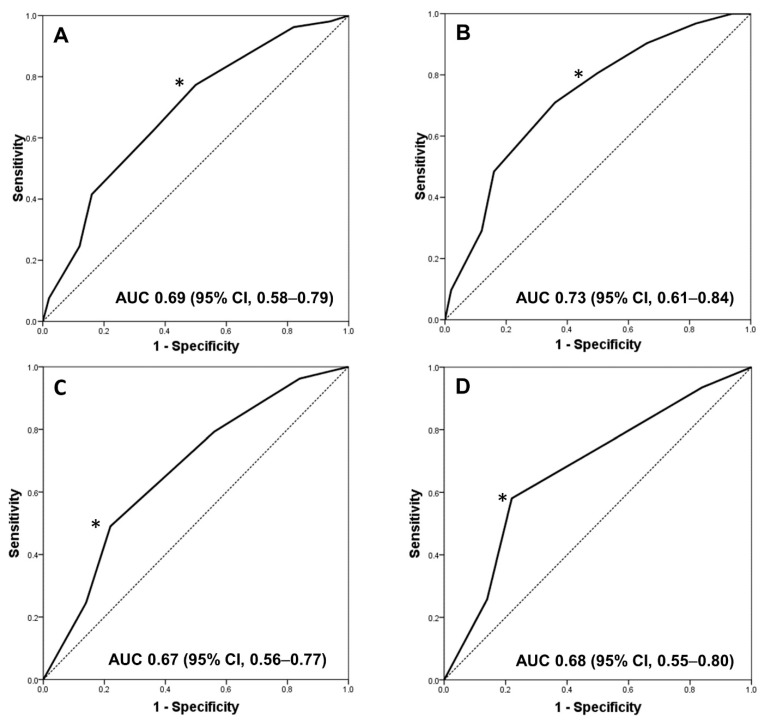
ROC curve for total number of HLA mismatches as a predictor of subclinical inflammation in the entire study population (**A**) and excluding patients with isolated mild inflammation (**B**). The optimal predictor cut-off value (*) was that of the highest sensitivity together with the lowest number of false positives (specificity). This value corresponds to 6 HLA-mismatches. (**C**,**D**) ROC curves for class II mismatches as a predictor of SCI in the entire population and excluding patients with isolated inflammation, respectively. The optimal predictor cut-off value (*) was 3 HLA mismatches.

**Figure 2:**
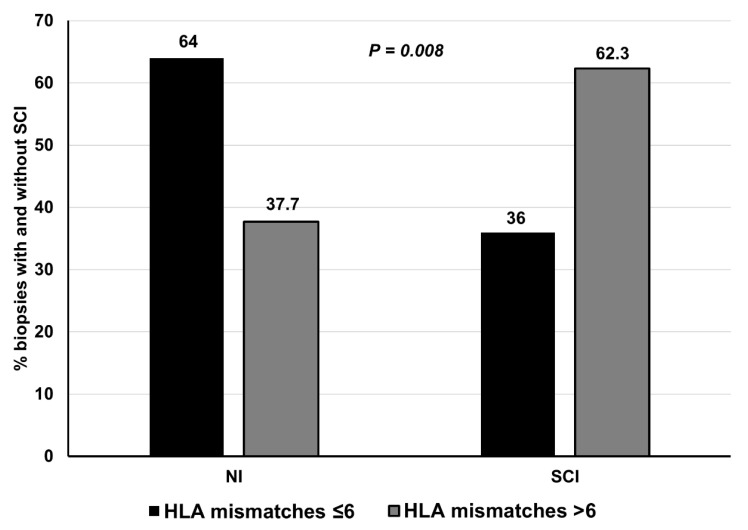
Proportion of biopsies performed on the third month post-kidney transplantation with and without subclinical inflammation in individuals with ≤6 vs. >6 HLA-mismatches. Abbreviations: NI, No inflammation; SCI, subclinical inflammation

Figure 1A shows the ROC curve for the total number of HLA mismatches and the SCI in the entire study population. The AUC for the number of HLA mismatches to predict SCI was 0.69 (95%CI, 0.58–0.79). The best Youden’s index corresponds with an HLA-mismatch cut-off value of 6, yielding a sensitivity of 77% and a specificity of 52% for SCI. An additional analysis excluding patients with IIF (*n* = 22) showed that an HLA-mismatch cut-off value of 6 had a sensitivity of 81% and a specificity of 51% for the clinical endpoint (AUC 0.73, 95%CI, 0.61–0.84) (Figure 1B). Similarly, the predictive performance of class II HLA mismatches in discriminating SCI in the entire population (Figure 1C) and excluding patients with isolated inflammation (Figure 1D) was 0.67 (95% CI, 0.56–0.77) and 0.68 (95% CI, 0.55–0.80), respectively, and three antigens was the optimal value in terms of sensitivity and specificity for predicting SCI. Eventually, when patients were clustered into two groups according to a cut-off value of 6 HLA-mismatches, a significantly higher proportion of SCI was observed in patients with >6 vs. ≤6 HLA mismatches (Figure 2). 

## 4. Discussion

This study demonstrates that HLA mismatching is independently associated with early SCI in low-immunological-risk KT recipients after adjusting for confounder variables. This harmful and unnoticed histological lesion in the absence of a protocol biopsy might lead to impaired renal function and graft loss in the long-term [8,9,32]. Accordingly, in daily clinical practice, transplant physicians should perhaps be more aware of HLA compatibility at the time of KT in order to avoid these unfavorable lesions. 

Recent years have witnessed controversy about the effect of HLA matching on kidney transplant outcomes because the development of potent immunosuppressants has allowed KTs of well- or poorly HLA-matched kidneys with similar success rates in terms of short- and medium-term survival [13,16,22,23,25,33]. However, the impact of HLA compatibility on early inflammatory lesions, especially SCI or borderline lesions, in the modern transplant era has been less well documented [10,12,17].

SCI exists in about 40–50% of allograft kidneys when protocol biopsies are performed routinely [5,34]. These lesions indicate an ongoing inflammation status that could perpetuate over time, evolving to chronic histological changes and allograft dysfunction [5,6,7,35,36]. In our study, the HLA mismatching score was an independent risk factor for early SCI (3rd month) despite the patients having a low-immunological risk at the time of KT. In addition, these patients also experienced a worse allograft function. Traditional alloimmune risk factors reported by KDIGO have been younger recipient age, panel reactive antibodies, transfusion prior to KT, cold ischemia time, or inadequate tacrolimus trough levels [37]. Our patients had panel reactive antibodies of zero, and other risk factors such as recipient and donor age, pretransplant transfusion, delayed graft function, induction therapy, or mean tacrolimus trough levels were not related with SCI in the multivariate analysis. The only risk factor associated with the appearance of early SCI was the total HLA-mismatch score. Although class II HLA DQ/DR mismatching was also independently associated with SCI in our study, as previously reported [18,25], an HLA antigen mismatch cut-off of 6, evidenced by ROC curve, proved to be the optimal predictor of SCI. As a result, patients with >6 HLA mismatches had a greater proportion of SCI compared with those with ≤6 HLA mismatches, suggesting an additive immune effect and likely a higher alloimmune risk category. It is plausible to think that the presentation of HLA class I and class II antigens leads to the activation and differentiation of the T cell into different cell subtypes (e.g., Th1, Th2, and Th17), initiating the early subclinical inflammatory phenomenon through the release of pro-inflammatory cytokines. Given that few data are available to support the prognostic importance of IIF on KT outcomes [9,36], we also performed a second analysis excluding patients with IIF, and again, we found that HLA mismatching was a strong risk factor for early SCI.

We speculate that the potential application of a proper HLA compatibility (e.g., <6 HLA mismatches) includes the administration of personalized immunosuppression in high-risk patients. For instance, older KT recipients with a longer dialysis vintage tend to exhibit an attenuated immune response. Thus, these patients could be treated less aggressively as long as a better HLA compatibility is achieved prior to KT in order to decrease life-threatening tumors or infections post-KT. On the other hand, in younger patients with a potentially increased immune response, the allocation of kidney donors with better HLA compatibility (HLA antigen >6) could help reduce the appearance of early inflammatory lesions in these patients. 

In agreement with our results, an independent correlation between HLA molecular mismatching and Banff borderline lesions has been recently demonstrated, suggesting that alloimmunity could be maintained over time in the presence of HLA antigen mismatches [17], leading to chronic histological lesions and impaired allograft function. In this regard, Williams et al., in a large cohort study, demonstrated via an elegant mathematical model a significant relationship between hazard ratios and HLA mismatches and allograft survival, highlighting the importance of HLA compatibility throughout the follow-up [15]. The fact that our patients with SCI had a higher chronicity score and greater proportion of IFTA, as well as worse allograft function, than those with NI supports this argument. 

This study had some limitations. First, we analyzed a relatively small sample of Caucasian low-immunological-risk KT recipients, and a larger study population might be required to support our findings. Additionally, we performed an association study, so no causation between HLA mismatching and SCI can be established. For these reasons, the results of our study may not be representative of other KT populations. However, major strengths of this study include the fact that we monitored our patients closely as part of a randomized clinical trial, and clinical and histological data were accurately collected for the entire study population, thus providing robustness to our findings. Secondly, we did not assess other HLA antigen mismatches (HLA-DP) or biomarkers for primary alloimmunity, such as HLA-DR/DQ single molecule eplet mismatch, which could improve the precision in primary alloimmune risk categorization [16,38,39]. Nevertheless, traditional HLA molecule mismatches used in our study are widely reproducible in daily clinical practice and have been known to correlate with transplant outcomes. Finally, we did not evaluate treatment adherence in our patients, though this was not within the scope of the study.

In conclusion, the HLA mismatch score is associated with early SCI in low-immunological-risk KT recipients. Although the strictly HLA-driven allocation of donor kidneys is not justified, because HLA is only one of many factors that influence KT outcomes, transplant physicians should perhaps be more aware of HLA compatibility in order to minimize these lesions, which may lead to graft loss in the long-term.

## Figures and Tables

**Table 1:** Baseline clinical data at three months post-transplantation.

	All *(n* = 105)	NI (*n* = 51)	SCI (*n* = 54)	*p* Value
Donor age (yr)	53.7 ± 12.6	52.4 ± 13.3	55.0 ± 11.8	0.311
ECD (%)	42.7	36.0	49.1	0.181
Recipient age (yr)	53.4 ± 12.4	53.5 ± 12.0	53.4 ± 12.9	0.980
Recipient BMI (kg/m^2^)	27.0 ± 4.4	28.0 ± 4.4	26.1 ± 4.3	0.030 *
Male (%)	73.3	72.5	74.1	0.860
Living donor (%)	14.6	16.0	13.2	0.688
Prior CVD (%)	19.4	18.0	20.8	0.724
Hemodialysis (%)	68.3	72.0	64.8	0.686
Cause of ESRD (%)				
Glomerulonephritis	21.0	17.6	24.1	0.357
Diabetes	16.2	23.5	9.3	
APKD	22.9	23.5	22.2	
Interstitial nephropathy	6.7	3.9	9.3	
Nephrosclerosis	9.5	11.8	7.4	
Unknown	12.4	9.8	14.8	
Other	11.4	9.8	13.0	
Induction therapy (%)				
Basiliximab	59	51	66.7	0.112
Thymoglobulin	41	49	33.3	
Transfusion prior KT	15.1	18.6	12.0	0.375
DGF (%)	25.5	24.0	26.9	0.735
Tacrolimus levels (ng/mL)	9.4 ± 2.5	9.7 ± 2.9	9.2 ± 2.1	0.286
Total HLA mismatches ^†^, median (IQR)	6 (5–8)	5.5 (4–7)	7 (6–8.5)	0.008 *
Class I HLA mismatches ^#^, median (IQR)	4 (3–5)	4 (3–5)	4 (3–5)	0.200
Class II HLA mismatches ^, median (IQR)	2 (1–3)	2 (1–2)	2 (2–3.5)	0.004 *
Proteinuria (mg/dL)	286.2 ± 224.9	297 ± 229	279 ± 225	0.763
Creatinine (mg/dL)	1.5 ± 0.4	1.4 ± 0.5	1.6 ± 0.4	0.018 *
MDRD-4 (mL/min)	54.1 ± 19.7	60.0 ± 23.4	48.5 ± 13.6	0.003 *

Abbreviations: APKD, adult polycystic kidney disease; BMI, body mass index; CVD, cardiovascular disease; ECD, expanded criteria donor; ESRD, end-stage renal disease; DGF, delayed graft function; HLA, human leucocyte antigen; KDPI, Kidney Donor Risk Index; KT, kidney transplantation; MDRD, modification of diet in renal disease 4 variable for estimating glomerular filtration rate at 3 months; NI, no inflammation at 3 months; SCI, subclinical inflammation at 3 months. Data are shown as the mean ± SD, median and interquartile range (IQR) and percentage. *p* value implies NI vs. SCI; ^†^ Includes HLA-A-B-C-DR-DQ mismatching; ^#^ Includes HLA-A-B-C mismatching; ^ Includes HLA-DR-DQ mismatching. * Statistically significant *p*-values.

**Table 2:** Multivariate logistic regression analysis for subclinical inflammation on the third month post-transplantation in the entire study population.

	OR	95% CI	*p* Value
Model 1			
Recipient age	1.00	0.96–1.04	0.924
DGF	1.53	0.55–4.26	0.414
Transfusion prior KT	0.68	0.21–2.26	0.530
Tacrolimus levels	0.89	0.75–1.07	0.212
Total HLA mismatches ^†^	1.32	1.06–1.64	0.013 *
Model 2			
Recipient age	1.00	0.97–1.04	0.964
DGF	1.53	0.56–4.21	0.409
Transfusion prior KT	0.73	0.22–2.47	0.614
Tacrolimus levels	0.89	0.75–1.06	0.192
Class II HLA mismatches ^	1.51	1.04–2.19	0.032 *

Included in the models, but not in the table, were donor age, induction therapy, cold ischemia time, class I HLA mismatches, and expanded criteria donor. Abbreviations: DGF, delayed graft function; KT, kidney transplantation; ^†^ Includes HLA-A-B-C-DR-DQ mismatching; ^ Includes HLA-DR-DQ mismatching. * Statistically significant *p*-values.

## Data Availability

Data available on request due to restrictions of privacy. The data presented in this study are available on request from the corresponding author. The data are not publicly available, due to incompliance with Organic Law 15/1999.

## Supplementary Materials

The following are available online at https://www.mdpi.com/article/10.3390/jcm10091934/s1, Table S1: Inflammatory and chronicity scores and clinical data in the NI and SCI groups at the baseline three-month protocol biopsy.
