# Supplementary material for: Impact of HLA Mismatching on Early Subclinical Inflammation in Low-Immunological-Risk Kidney Transplant Recipients
Source: J Clin Med. 2021 Apr 29;10(9):1934. doi: 10.3390/jcm10091934 (PMC8125522; doi:10.3390/jcm10091934)
Supplement: Supplementary file 1 [file jcm-10-01934-s001.zip › jcm-1170042-supplementary.pdf]

Table 1. Inflammatory and chronicity scores and clinical data in the NI and SCI groups at the baseline three-month protocol biopsy.

	**NI (*n* = 51)**	**SCI (*n* = 54)**	***p* value**
**g (1–3)**	2.2	9.3	0.137
**ptc (1–3)**	0	19.2	0.001 *
**t (1–3)**	0	59.3	0.000 *
**i (1–3)**	0	100	0.000 *
**v (1–3)**	0	2.0	0.352
**ci (1–3)**	28.9	58.0	0.004 *
**ct (1–3)**	25.0	51.0	0.010*
**cg (1–3)**	0	0	-
**cv (1–3)**	27.3	46.9	0.051
**ah (1–3)**	22.9	33.3	0.244
**ct+ci**	27.3	60.4	0.001 *
**IFTA ≥2**	25	45.8	0.037 *
**ct+ci+cg+cv**	42.9	75.0	0.002 *

Abbreviations: ah, arteriolar hyaline thickening; ci, chronic interstitial fibrosis; cg, transplant glomerulopathy; ct, chronic tubular; cv, fibrous intimal thickening; g, glomerulitis; i, interstitial infiltration; IFTA, proportion of patients with sum of interstitial fibrosis and tubular atrophy; NI, no inflammation; ptc, peritubular capilaritis; SCI, subclinical inflammation; t, tubulitis; v, arteritis. Data are shown as percentage. *statistically significant p-values.
